# Cases of Pins Extracted from the Breast of a Woman, after Remaining There Sixty Years

**Published:** 1797

**Authors:** Henry Fryer

**Affiliations:** Surgeon at Stamford, in Lincolnshire.


Page(s) missing

Page(s) missing down ſtairs upon her face, by which fall ſhe had hurt her breaſt, and then firſt acknowledged that ſhe had, many years before, thruſt ſeveral pins into her breaſts. Upon examination I found I could very readily ſhake about a great number of pins, (which ſeemed of different ſizes, as well as ſhapes) not only in each breaſt, but alſo in the ſkin upon the ſcrobiculus cordis.

On farther inquiry I learned, that about ſixty years ago, when between fifteen and ſixteen years old, ſhe had been in ſome degree deranged, and had at that time forced theſe pins into the ſkin, but had never felt any inconvenience from them till now, when ſhe had fallen down; and, I ſuppoſe, ſome of the pins being preſſed hard againſt the ſkin, as they lay between the ſkin and the ribs, with ſcarcely any adipoſe ſubſtance intervening, had occaſioned the pain. She was perfectly rational at this time, and gave me the above account herſelf.

As ſhe pointed readily to thoſe pins which gave her pain, I made a ſmall opening with a lancet, and took out three, one crooked, the others ſtraight; they were turned very black, and I had ſome difficulty in detaching them. I ſaw her again in a few days, when the wound was was nearly healed, but ſhe complained then of ſome others; I therefore made another ſmall opening, and removed two more pins, after which ſhe would not conſent to have any more taken away, as ſhe ſaid that ſhe then felt no pain from them.

The number of pins ſtill remaining I can by no means gueſs at; but it is ſo conſiderable, that I can feel them ſtrike againſt each other upon taking hold of either of her breaſts, particularly as her ſkin hangs looſely from her, ſhe being now very thin; and alſo upon laying my hand upon the ſkin between her breaſts, I can feel them in lumps, lying in every direction.

By way of poſtſcript to this extraordinary proof of the little trouble that extraneous bodies will ſometimes occaſion, I will add the following:

In the year 1787 I took out of a man's leg a thorn, nearly two inches long, which had lain there twenty-two years, without giving him any pain; and after it was taken out the wound healed in two or three days without any trouble. The thorn lay lengthways on the outer edge edge of the gaſtrocnemius, near the upper part of that muſcle.

*Stamford,*

*October 6, 1795.*

